# *In vivo* recording of aerodynamic force with an aerodynamic force platform: from drones to birds

**DOI:** 10.1098/rsif.2014.1283

**Published:** 2015-03-06

**Authors:** David Lentink, Andreas F. Haselsteiner, Rivers Ingersoll

**Affiliations:** Department of Mechanical Engineering, Stanford University, Stanford, CA 94305-3030, USA

**Keywords:** aerodynamic, force platform, *in vivo*, non-intrusive, control volume, bird

## Abstract

Flapping wings enable flying animals and biomimetic robots to generate elevated aerodynamic forces. Measurements that demonstrate this capability are based on experiments with tethered robots and animals, and indirect force calculations based on measured kinematics or airflow during free flight. Remarkably, there exists no method to measure these forces directly during free flight. Such *in vivo* recordings in freely behaving animals are essential to better understand the precise aerodynamic function of their flapping wings, in particular during the downstroke versus upstroke. Here, we demonstrate a new aerodynamic force platform (AFP) for non-intrusive aerodynamic force measurement in freely flying animals and robots. The platform encloses the animal or object that generates fluid force with a physical control surface, which mechanically integrates the net aerodynamic force that is transferred to the earth. Using a straightforward analytical solution of the Navier–Stokes equation, we verified that the method is accurate. We subsequently validated the method with a quadcopter that is suspended in the AFP and generates unsteady thrust profiles. These independent measurements confirm that the AFP is indeed accurate. We demonstrate the effectiveness of the AFP by studying aerodynamic weight support of a freely flying bird *in vivo*. These measurements confirm earlier findings based on kinematics and flow measurements, which suggest that the avian downstroke, not the upstroke, is primarily responsible for body weight support during take-off and landing.

## 1. Introduction

Animals that fly with flapping wings range from insects and bats to birds. The latter have complex wing morphologies and motions, which affect their ability to generate aerodynamic force in ways that are not fully understood [1]. The current method to measure the aerodynamic force of a flapping wing directly is to tether an animal or robot and measure the forces transferred through the tether with a load cell [2–4]. Tethered experiments with animals raise obvious concerns, but even tethered robot experiments are inaccurate when confounding inertial forces cannot be accounted for through dynamic modelling or measurement [5]. Whereas tethered experiments have been the primary solution for evaluating aerodynamic force, measurements during free flight manoeuvres are intrinsically more informative. During free animal movement, aerodynamic force can be calculated non-intrusively in three ways. First, the shape and motion of small animals, particularly insects, can be measured to compute the flow fields and forces using computational fluid dynamics [1,6]. Second, the body mass and acceleration distribution can be measured and integrated using dynamics models to calculate force; this method requires sacrificing animals after their body kinematics have been measured [7,8]. Finally, the airflow can be measured around the animal and integrated using (simplified versions of) the Navier–Stokes equations to calculate the net aerodynamic force [9–17]. All these calculations are based on indirect measurements of variables that need to be differentiated or integrated numerically to calculate force, which introduces numerical error. A non-intrusive, real-time, direct force measurement method (similar to the instrumented tether) does not exist for studying free locomotion in fluids. For studying terrestrial locomotion such a solution does exist: the force platform [18]. Here, we present an aerodynamic force platform (AFP) that enables such measurements in fluids. We first justify the new method with an analysis using the Navier–Stokes equations, then validate it with a tethered quadcopter, and finally we demonstrate *in vivo* recordings for a freely flying bird.

## 2. Fluid-mechanical analysis

**Figure 1.**
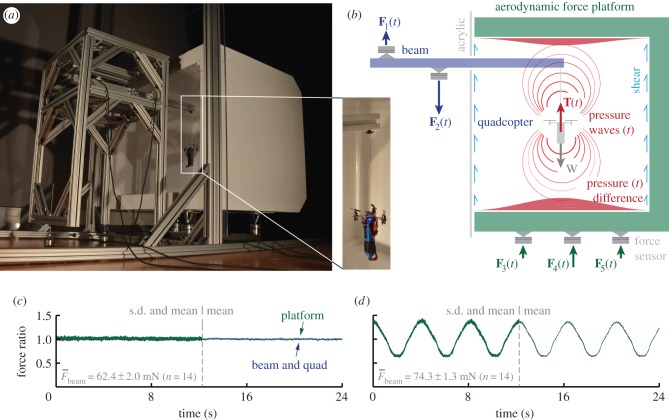
Aerodynamic force platform (AFP) working principle. (*a*) Validation of the platform: an overweight quadcopter, hung from a beam instrumented with load cells, suspended in the AFP (an instrumented box). The inset shows a close-up of the quadcopter and its elongated battery—too heavy to take-off. (*b*) Based on Newton's third law, the thrust force of the quadcopter, **T**, is balanced by the beam's support forces: **F**_1_ − **F**_2_. The thrust force, **T**, is transferred to air, which transfers it as a pressure force normal (and a small shear force tangential) to the walls of the AFP, resulting in the ground reaction force: **F**_3_ + **F**_4_ + **F**_5_. Pressure waves transfer fluctuations in the thrust force at the speed of sound to the surrounding air, and ultimately the platform. The validation is carried out by generating constant (*c*) and sinusoidal (*d*) thrust profiles with the quadcopter, which are measured with the platform (green) versus beam (blue). Both thrust measurements are normalized with the time-average thrust measured by the beam, which gives the force ratio. Both the standard deviation (left) and mean (right) traces overlap, which demonstrates that the platform is accurate. (Online version in colour.)

The AFP is a box, instrumented with load cells, that encloses the object or animal that generates the net unsteady fluid force (figure 1*a*). It works based on Newton's third law applied to a fluid; the unsteady net fluid force needs to be supported by an equal and opposite net force that acts on the control volume boundary. The AFP is thus a mechanical representation of the control surface integral of the Navier–Stokes equation [19] that calculates the net time-dependent force2.1
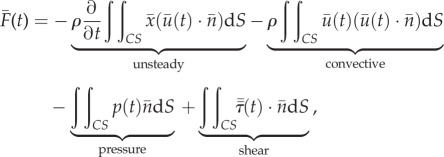
where *ρ* is density, 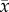 is position, 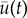 is velocity, d*S* is the integration surface, 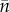 is the surface normal vector, *p*(*t*) is pressure and 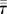 is the shear stress tensor. This net fluid force can be integrated exactly, provided that the three-dimensional velocity, the pressure and shear stress are known over the complete control surface as a function of time. In addition, small phase differences owing to the finite propagation speed of pressure waves (sound) must be small [20], which is the case when the ‘AFP number’ of the control volume is much smaller than one2.2
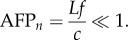
This condition is met when the control volume has an order of magnitude smaller length scale, *L*, than the distance sound travels, at speed *c*, within the shortest period of interest (1/*f*; in which *f* is the frequency) that needs to be resolved in the fluid force 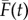 . Under this condition, the control surface integral (2.1) can be accurately evaluated. In this study, the largest AFP*_n_* ≤ 0.022 (*L* ≤ 0.41 m, *f* ≤ 18 Hz, *c* = 340 m s^−1^), which implies that the aerodynamic phase delay is of order 2% compared with the wingbeat time of the parrotlets we study.

Whereas the unsteady and convective terms are significant in the bulk fluid, they vanish at the surface of the AFP. The contour integral is simplified by substituting the no-slip and no-flow condition on the surface of the physical control surface2.3
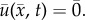
This gives the following surface integral for the net fluid-dynamic force, which depends on the pressure and shear stress distribution that act on the surface of the AFP:2.4
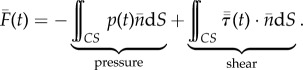
The pressure and shear stress at the surface are time-dependent and driven by the flow in the volume. In theory, it is essential that the entire control surface is a rigid enclosure, formed by walls, in order to guarantee that the net fluid force is measured accurately (figure 1*b*). In practice, however, viscous shear forces acting on the wall can typically be ignored, compared with pressure forces if the Reynolds number is much larger than unity [21]2.5
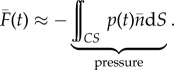
Finally, the net acceleration of the moving object or animal can be calculated by dividing the net measured fluid force minus body weight by the net associated mass2.6
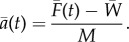


## 3. Experimental validation

**Figure 2.**
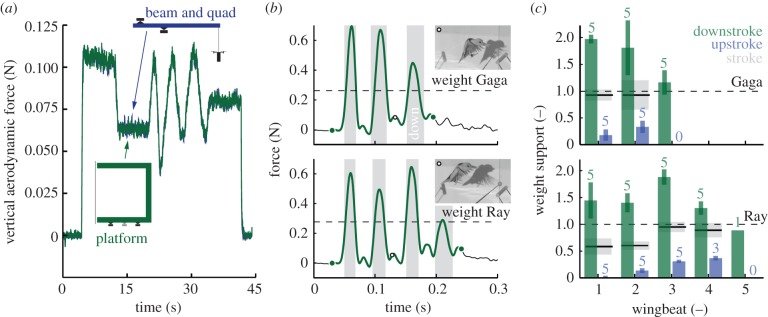
The aerodynamic force platform measures weight support of a quadcopter and freely flying birds *in vivo*. (*a*) The quadcopter's unsteady thrust measured with the platform (green) versus beam (blue) overlap, confirming that the platform is accurate (fourth-order Butterworth filter with 30 Hz cut-off for AFP and beam). (*b*) Force-platform measurements of two Pacific parrotlets (Gaga and Ray) flying between two perches at 0.28 m distance in the AFP (fourth-order Butterworth filter with 60 Hz cut-off; green circle, take-off and landing; circle with black outline, video frame; grey area, downstroke). The snapshots illustrate that the feathers open the wing surface like a venetian blind during the upstroke. (*c*) Calculation of wingbeat-averaged weight support based on raw data (flights, *n* = 5; birds, *N* = 2). During take-off, Ray pushes off more vertically than Gaga, as illustrated in the electronic supplementary material videos. The start of the downstroke and upstroke is defined as the moment when the wing is at its highest and lowest position, for the last wingstroke(s) we evaluate stroke direction. (Online version in colour.)

We developed an AFP, calibrated it, measured its natural frequency and validated it using independent load cell measurements on a tethered quadcopter (figures 1 and 2*a*). To obtain the net unsteady force with a mechanical implementation of the contour surface integral, the walls need to be instrumented with load cells that measure the net fluid forces (and moments). Similar to terrestrial force platforms [18], the natural frequency of the instrumented walls of the AFP needs to be an order of magnitude greater than the highest-frequency force fluctuation of interest (this requires high stiffness and lightweight design). Further, the sensors need sufficient sensitivity to detect the smallest forces and sufficient dynamic range to resolve the largest forces.

Our first-generation AFP is essentially a lightweight and stiff instrumented box with one open side for easy access that is covered with an acrylic plate (figure 1*a*). The walls are made out of thin (1 mm) balsa wood sheets that are combined into a sandwich structure that maximize platform stiffness with respect to weight (outer height × width × depth: 0.530 × 0.634 × 0.507 m; inner: 0.420 × 0.525 × 0.452 m). The box is supported by three Nano 43 sensors (six-axis, with SI-9-0.125 calibration, ATI Industrial Automation) that sample force at 1 ms intervals with 2 mN resolution. To precisely resolve vertical force, the AFP is connected statically determined (moment free) to the three load cells. The sensors are arranged such that all are equally preloaded by AFP weight (1.79 kg; linear calibration coefficient: 0.989; *r*^2^ = 1.000, rounded to three decimals). The natural frequency was measured by popping a balloon five times near the platform. The natural frequency in the vertical (thrust) direction is 132 Hz, which is weakly coupled to a small-amplitude 105 Hz mode.

To validate and evaluate the accuracy of the AFP, we need independent, ground-truth, aerodynamic force measurements. For this, we attached a quadcopter (Estes 4606 Proto X Nano, with a 1500 mAh Li–Po battery) to an instrumented aluminium beam with a Kevlar tether (two Nano 43 sensors; natural frequency beam + quadcopter, 138 Hz; linear calibration coefficient: 0.997; *r*^2^ = 1.000, rounded). Visual comparison of the beam versus AFP measurement suggests that the correspondence in force measurement is remarkably close for a hand-controlled thrust profile (figure 2*a*). For systematic validation, we modified the RC control of the quadcopter to transmit semi-sinusoidal and constant thrust profiles using an Arduino Uno microcontroller (figure 1*c,d*). All measured quadcopter thrust profiles (AFP and beam) were filtered using a fourth-order Butterworth filter with a cut-off frequency of 30 Hz (Matlab R2010a).

Comparison of the impulse and force ratio of the thrust profiles measured with the AFP versus beam shows that our AFP is accurate to within 2%—which is equivalent to the load cell resolution limit of 2 mN (table 1). Using cross-correlation, we find the time delay of the AFP is within its natural vibration period. This time delay, owing to the transfer function of the AFP, is an order of magnitude larger than the delay owing to the speed of sound (about 1 ms), showing that the delay is primarily determined by the AFP's structural dynamics. The 2% higher force measured by the AFP is equivalent to the missing shear force that should act on the acrylic plate, which is not instrumented, assuming the Blasius equation for boundary layer friction of a flat plate [22]. Our back-of-the-envelope estimate of the net shear force, for a measured near-wall flow speed of 0.95 ms^−1^ (with a hotwire) at 25°C, gives a shear force equal to +0.8% of the total force, below the load cells' resolutions, but in the right direction. This supports our notion that we can probably ignore shear at Reynolds numbers much greater than unity (*Re* ≈ 27 000 based on AFP height), which can greatly simplify future AFP design. Comparative thrust measurements using an acrylic front plate with and without a circular gap further demonstrate that shear force can be ignored. These experiments show that the gap can be larger than 20% surface area without affecting measurement accuracy. This enables simple validation experiments and interaction with animals flying in the AFP. Finally, we note that the AFP is a special kind of infrasound microphone; for our AFP, we calculated a sensitivity of up to 0.008 Pa. We found that high sensitivity requires elimination of all infrasound noise sources in the laboratory; this includes switching off air conditioners because they can increase noise by an order of magnitude. We also found that if the AFP is installed statically determined, it does not require special vibration isolation measures.

**Table 1.** Validation of AFP versus beam measurement of integrated impulse and instantaneous force of a quadcopter shows that the AFP is accurate and time-resolved.

experiment	total impulse ratio (−)	ave. force ratio (−)	delay (ms)
constant (*n* = 14)	1.016 ± 0.011	1.017 ± 0.011	—
0.125 Hz (84 periods)	1.014 ± 0.006	1.014 ± 0.006	2 ± 2
0.250 Hz (84 periods)	1.010 ± 0.006	1.011 ± 0.006	6 ± 1
0.500 Hz (84 periods)	1.017 ± 0.004	1.018 ± 0.005	8 ± 1

## 4. *In vivo* demonstration and outlook

To demonstrate that the AFP can directly measure the aerodynamic force generated by a freely flying animal, we trained two Pacific parrotlets (*Forpus coelestis*; 28 g; 0.2 m wingspan; wingbeat frequency 20 Hz) to fly between two perches in the AFP. The perches were connected to the acrylic front panel. To enable training and cueing and rewarding of the bird, we made a gap in the acrylic front panel of 0.071 m^2^, which has no measurable effect on accuracy (table 2). Each parrotlet was trained using habituation and positive reinforcement [23–25], based on millet seed rewards to fly to a target stick and touch it with its beak (food and water ad libitum; cages have enrichment, animals were not sacrificed, all training and experimental procedures were approved by Stanford's Administrative Panel on Laboratory Animal Care). Its aerodynamic weight support was measured *in vivo* within a wingbeat using the AFP, whereas the start and end of the wingbeat were determined with a synchronized high-speed camera at 1000 fps (Phantom Miro M310; figure 2*b*). We selected a 60 Hz low-pass cut-off frequency to avoid interference with the wing beat frequency. The recordings demonstrate that the upstroke of generalist birds, such as the parrotlet, does not aerodynamically support body weight (much) during take-off and landing manoeuvres (figure 2*c*; irrespective of interspecific differences in flight style). Instead they generate a vertical force of up to twice their body weight during the downstroke. This direct force measurement validates earlier indirect force estimates based on kinematic [7,8], flow [12,13,26,27] and *in vivo* pressure measurements [28,29], which indicate that the avian upstroke produces little weight support during slow flight [30].

**Table 2.** A hole in one of the sidewalls of the AFP has no effect on vertical impulse and force accuracy (average of five recordings of 6 s each).

diameter hole (m)	area ratio (−)	total impulse ratio (−)	ave. force ratio (−)
0	0	1.019 ± 0.004	1.019 ± 0.004
0.100	0.036	1.020 ± 0.011	1.020 ± 0.011
0.175	0.109	1.018 ± 0.003	1.018 ± 0.003
0.250	0.223	1.021 ± 0.003	1.021 ± 0.003

The capability of the AFP to measure aerodynamic force *in vivo* is applicable across taxa and addresses the welfare of experimental animals; it is non-invasive, no-touch and thus relatively low-stress. Future AFPs can be improved by constructing them using sandwich structures consisting of carbon fibre and Nomex honeycomb. Optical access can be improved using tensioned transparent membranes. The measurement sensitivity can be increased with custom load cells that harness extremely precise capacitive or interferometric displacement sensors. Ultimately, the three-dimensional force vector can be resolved with a fully enclosing AFP. This approach can be extended to much larger volumes by composing the side walls out of individually instrumented plates to obtain higher natural frequencies, which also allows for decomposition of force components. The mechanical design of the platform can be scaled down for insects to achieve higher sensitivities and natural frequencies. Scaling up or down involves straightforward lightweight structural design [31] for appropriate natural frequencies [31,32] of the AFP and the load cells, similar to the design of a terrestrial force plate [18]. The main difference between an AFP and terrestrial force plate is that AFPs are much more sensitive to pressure per unit surface area. This sensitivity also makes the AFP applicable to wind tunnels in which they could integrate the wall pressure distribution to determine the vertical aerodynamic force [33]. Theoretically, this real-time method should work for many animals, robots and objects that generate a net force in a fluid. Both vertebrates and invertebrates can be trained to behave in the AFP using habituation and operant conditioning [23–25,34]; alternatively, an attractive food source can be placed in the AFP to work with untrained animals in their natural habitat. Here, we have already demonstrated that AFPs can be used to evaluate the aerodynamic force generation of drones *non-intrusively*, and freely flying birds *in vivo*, with remarkable precision.
